# Juvenile idiopathic arthritis is associated with reduced levels of biochemical bone status indices reflecting altered bone formation

**DOI:** 10.1186/s41927-026-00710-3

**Published:** 2026-11-03

**Authors:** Per Lewander, Diana Atanasova, Charlotte Dahle, Jonas Wetterö, Christopher Sjöwall, Per Magnusson

**Affiliations:** 1 Clinical Department of Paediatrics in Norrköping, Region Östergötland, Norrköping, Sweden; 2 Department of Biomedical and Clinical Sciences, Linköping University, Linköping, Sweden; 3 Department of Clinical Chemistry, Region Östergötland, Linköping, Sweden; 4 Department of Clinical Immunology and Transfusion Medicine, Region Östergötland, Linköping, Sweden

**Keywords:** Biomarkers, Bone, Bone mineral density, Alkaline phosphatase, Cartilage, C-terminal telopeptide of type I collagen, Digital radiography, Juvenile idiopathic arthritis, Procollagen type I N-propeptide, Type II procollagen C-terminal propeptide

## Abstract

**Introduction:**

Individuals with juvenile idiopathic arthritis (JIA) are at increased risk of impaired bone health and fractures. This prospective case-control study assessed bone mineral density (BMD) and bone and cartilage biomarkers in children with JIA and healthy controls.

**Methods:**

Fifty-one individuals with well-characterized JIA (age 1.6–17.9 years) and 61 age-matched controls from the same region were included. Joint erosions were assessed radiographically, and BMD was measured by digital X-ray radiogrammetry (DXR). Bone formation was assessed using serum intact procollagen type I N-propeptide (PINP) and bone-specific alkaline phosphatase (BALP), and bone resorption was assessed by C-terminal telopeptide of type I collagen (CTX). Cartilage synthesis was assessed by serum type II procollagen C-terminal propeptide (PIICP). Biomarkers were measured at baseline and 1-year follow-up, while joint erosions were evaluated up to 5 years. Soluble urokinase plasminogen activator receptor (a.k.a. suPAR), rheumatoid factor, anti-cyclic citrullinated peptide antibodies, and antinuclear antibodies were also measured.

**Results:**

The DXR Z-scores were lower in the polyarticular subtype JIA group in comparison with controls (*p* = 0.010). The polyarticular JIA group had lower PINP levels in comparison with controls at inclusion (*p* = 0.028) and after 1 year (*p* = 0.006). Serum BALP levels were lower in the overall JIA group and in the polyarticular group at 1 year, *p* = 0.031 and *p* = 0.042, respectively. Plasma CTX levels were lower in individuals receiving oral steroids compared with those not receiving steroid therapy (*p* = 0.006). Higher PIICP levels were found in the oligoarticular subtype JIA group in comparison with the control group (*p* = 0.020).

**Conclusions:**

Based on our findings, children with JIA, particularly those with polyarticular disease, are at increased risk of compromised bone health over time, potentially related to reduced bone formation. These findings underscore the importance of monitoring bone health and implementing targeted interventions to promote optimal peak bone mass acquisition in children with JIA.

## Introduction

Juvenile idiopathic arthritis (JIA) is a heterogeneous group of inflammatory joint disorders with onset before 16 years of age [1]. Typical symptoms include joint pain, swelling and stiffness, and in some cases fever, swollen lymph nodes, rash and fatigue [2–5]. The clinical course of JIA varies considerably; it can be non-destructive and self-limiting, but up to 60% of individuals may develop a chronic, progressive form that can lead to permanent joint damage [6]. Predictors of worse outcomes include polyarticular involvement, specific joint involvement (ankle, wrist, and cervical spine), delayed diagnosis, presence of anti-cyclic citrullinated peptide antibodies (anti-CCP) and rheumatoid factor (RF), and delayed initiation of treatment [7, 8].

In addition to the primary symptoms arising from the inflammatory processes in the joints, JIA is associated with several comorbidities affecting multiple organs that cause severe complications of body tissues [5, 9]. Individuals with JIA may develop low bone mineral density (BMD) at a young age, which increases the risk of fractures throughout life [4, 10, 11]. Vertebral fractures are particularly common in polyarticular and systemic JIA subtypes, and the use of systemic glucocorticoids further increases the risk of both vertebral and non-vertebral fractures [12, 13].

Dual-energy X-ray absorptiometry (DXA) is the gold standard for BMD assessments. Besides excellent precision and reproducibility, DXA provides whole body and local (forearm, hip, and lumbar spine) measurements of BMD. Digital X-ray radiogrammetry (DXR) is a less expensive alternative to DXA for BMD measurements [9, 10, 14]. The DXR measurements are based on a standard radiograph of the hand, from which an estimation of BMD is automatically calculated from a single radiograph. It has been reported that DXR measurements offer a BMD estimate with low variation between local sites, are less prone to movement artifacts, and have a good association with distal forearm BMD [12, 15, 16]. The use of DXR has been approved by the U.S. Food and Drug Administration for BMD estimation [17]. As with DXA measurements, DXR-derived BMD Z-scores can be calculated from healthy reference populations of the same age and sex. Taken together, DXR is a convenient and cost-effective method for evaluating BMD in pediatric populations with an increased fracture risk.

Bone tissue is continuously remodeled throughout life by a carefully orchestrated process through bone resorption by osteoclasts and bone formation by osteoblasts. Bone modeling is another unique process that occurs predominantly during skeletal growth, where bones are shaped or reshaped by the independent action of osteoclasts and osteoblasts. Biochemical markers of bone turnover reflect the cellular processes of modeling and remodeling. Bone formation can be assessed in the circulation by intact procollagen type I N-propeptide (PINP) and bone-specific alkaline phosphatase (BALP). Serum PINP reflects type I collagen synthesis, the first phase of osteogenesis, whereas BALP reflects extracellular matrix maturation prior to the initiation of mineralization [18]. Bone resorption can be assessed by C-terminal telopeptide of type I collagen (CTX), which measures type I collagen degradation [19]. Type II procollagen C-terminal propeptide (PIICP) reflects the extent of joint cartilage biosynthesis [20]. The interpretation of biomarkers of bone turnover is more complex in children and adolescents since they can have 5- to 20-fold higher levels in comparison with adults due to contributions from pubertal growth [21, 22].

Despite their clinical relevance, studies on bone mass and biomarkers of bone and cartilage remodeling in JIA remain limited. Additional studies are therefore warranted to elucidate mechanistic pathways and to guide interventions for optimizing bone health in individuals with JIA [23, 24]. Biomarkers associated with an aggressive disease course are of particular interest to individualize treatment strategies. Findings from our previous study indicate that, in addition to RF and anti-CCP, serum soluble urokinase plasminogen activator receptor (suPAR, same as soluble CD87) could be of additional value in assessing the risk of joint erosions in children with JIA [25].

This prospective case-control study was designed to investigate bone mass by DXR-BMD, biomarkers of bone and cartilage remodeling in a well-characterized cohort of individuals with different subtypes of JIA. These outcomes were evaluated in relation to anti-CCP, RF, antinuclear antibodies (ANA), and suPAR. All biomarkers were assessed at inclusion and after 1 year, while clinical evaluations of joint erosions were followed for up to 5 years.

## Materials and methods

### Subjects and study design

This prospective case-control study enrolled children and adolescents with JIA classified according to the International League of Associations for Rheumatology criteria were included [1]. Children and adolescents aged 0–18 years with newly diagnosed JIA or established JIA requiring regular clinical follow-up were eligible for inclusion. Patients were excluded if they had traumatic joint involvement or concomitant conditions known to affect bone metabolism, growth, or skeletal development, including endocrine, metabolic, gastrointestinal, or renal disorders. All participants were followed regularly at the Department of Pediatrics, Vrinnevi Hospital in Norrköping, Sweden, by the same pediatrician (author P.L.). The presence of joint swelling, tenderness, or motion-induced joint pain, without traumatic origin, were evaluated according to the American College of Rheumatology definition of disease activity [26]. Clinical information regarding JIA subtypes (oligoarticular, polyarticular, juvenile psoriatic arthritis (JPsA), enthesitis-related arthritis (ERA), undifferentiated arthritis, and systemic JIA), and disease-modifying anti-rheumatic drugs (DMARDs), including biological therapies, were documented and followed over a 5-year period.

The control group were recruited among friends and siblings to the individuals with JIA. Exclusion criteria were arthritis, rheumatic disease, asthma, celiac disease, kidney disease, hypertension, and bone and mineral metabolic diseases. Except for the DXR measurements, the joints of control subjects were not examined using X-ray radiography, and they were only sampled at baseline. The study was approved by the Regional Ethics Committee at Linköping University (M70-7/2007) and was performed in accordance with the Declaration of Helsinki. Oral and written informed consents were obtained from all participants and their caregivers.

### Radiography and assessment of DXR-BMD

Conventional X-ray radiography investigations of affected joints were performed and evaluated at the Department of Radiology, Vrinnevi Hospital, Norrköping, Sweden, but not necessarily by the same radiologist. Radiographs were acquired as part of routine clinical care using radiographic systems subject to established quality assurance programs, including regular technical performance testing, phantom-based image quality assessments, monitoring of radiation dose parameters, and evaluation of acquisition quality. Only radiographs of sufficient technical quality for DXR-BMD analysis were included. Assessments of BMD were performed by DXR (Sectra AB, Linköping, Sweden) on a standardized X-ray of the left hand on each individual with JIA as reported elsewhere [9]. Z-scores of DXR-BMD were calculated from age- and sex-specific reference intervals based on data from a Dutch Caucasian pediatric cohort of 535 healthy children and adolescents [9].

### Biochemical determinations

Blood samples were collected from all study participants and aliquots were stored at − 70 °C until analysis. The erythrocyte sedimentation rate (ESR) and serum C-reactive protein (CRP) were analyzed at the Department of Clinical Chemistry (Swedac accredited no. 1342), Vrinnevi Hospital, Norrköping, Sweden. Serum CRP was determined by turbidimetry on an ADVIA^®^ 1800 analyzer (Siemens Healthcare Diagnostics, Terrytown, NY, USA). Concentrations of CRP < 10 mg/L were reported as 5 mg/L.

Serum ANA, RF and anti-CCP were analyzed at the Department of Clinical Immunology and Transfusion Medicine (Swedac accredited no. 1342), Linköping University Hospital, Linköping, Sweden. Indirect immunofluorescence microscopy using slides with fixed HEp-2 cells (Immuno Concepts, Sacramento, CA, USA) and fluorescein isothiocyanate conjugated γ-chain-specific anti-human IgG as detection antibody (Agilent, Glostrup, Denmark) was used for ANA analyses, as reported elsewhere [25]. Serum RF was analyzed by nephelometry (Beckman Coulter, Inc., CA, USA), and anti-CCP by fluorescence enzyme immunoassay on a Phadia 250 instrument (EliA™, Thermo Fischer Scientific, Uppsala, Sweden). Serum suPAR was measured by the suPARnostic^®^ AUTO Flex enzyme-linked immunosorbent assay (ELISA) (ViroGates A/S, Birkeroed, Denmark) as reported elsewhere [25].

Serum intact PINP was assessed with the UniQ radioimmunoassay (Aidian Oy, Espoo, Finland), with an analytical range 5–250 µg/L, intra-assay CV of < 5%, and interassay CV of < 6%. Serum BALP was measured by the MicroVue™ BAP ELISA (Quidel Corp., San Diego, CA), analytical range 0.7–140 U/L, intra-assay CV of < 6%, and interassay CV of < 8%. Circulating CTX was measured in EDTA plasma samples by the CrossLaps^®^ ELISA (Immunodiagnostic Systems Holdings PLC, Boldon, UK), analytical range 20–3380 ng/L, intra-assay CV of < 6%, and interassay CV of < 10%. Serum PIICP was measured by ELISA (EKX-IPR66X-96, Nordic Biosite, Täby, Sweden), analytical range 31–2000 ng/L, intra-assay CV of < 8%, and interassay CV of < 10%.

### Statistical analysis

The Shapiro-Wilk test was used to analyze the normal distribution of data, and the Mann Whitney U test was used to compare biomarker levels between the JIA and control groups, as well as between the JIA subtypes. Differences in sex distribution between groups were assessed using Fisher’s exact test (two-sided). Correlations were assessed by Spearman’s rank correlation coefficient. *P*-values of less than 0.05 were considered significant. All analyses were performed using GraphPad Prism version 10.6.1 (GraphPad Software Inc., La Jolla, CA, USA).

## Results

Fifty-one children and adolescents, aged 1.6–17.9 years, comprising 32 females and 19 males, with JIA were included in this prospective case-control study. The JIA subtypes were as follows: oligoarticular arthritis (*n* = 21), polyarticular (*n* = 17) including RF positive and RF negative, (*n* = 4 and *n* = 13, respectively), JPsA (*n* = 5), ERA (*n* = 4) and undifferentiated arthritis (*n* = 4), and none was diagnosed with systemic JIA. Disease duration varied between 0.1 and 13.4 years, with a median of 1.6 years at inclusion. According to the American College of Rheumatology definition of disease activity [26], 46 individuals (90%) had “active disease” at inclusion, and 10 of them were newly diagnosed (within 6 months). The control group comprised 61 age-matched healthy children and adolescents, 28 females and 33 males, aged 2.0–18.1 years. There were no statistically significant differences in age or sex distribution between the JIA and control groups. Clinical characteristics at study start are presented in Table 1.

**Table 1:** Characteristics and demographics of the JIA and control groups at study start

	JIA (*n* = 51)	Controls (*n* = 61)	*P*-value
Age at enrolment (years)	12.9 (1.6–17.9)	13.3 (2.0–18.1)	0.690
Sex (Females)	32 (63%)	28 (46%)	0.090
*JIA subtypes:*			
Oligoarticular Polyarticular, RF positive Polyarticular, RF negative JPsA ERA Undifferentiated Systemic	21 (41%) 4 (8%) 13 (26%) 5 (10%) 4 (8%) 4 (8%) 0		
Disease duration at inclusion (years)	1.6 (0.1 − 13.4)		
C-reactive protein (mg/L)	9 (5–40)		
Erythrocyte sedimentation rate (mm/h)	10 (2–34)		
ANA+	22 (43%)		
RF+	5 (10%)		
Anti-CCP+	6 (12%)		
RF + and anti-CCP+	4 (8%)		
NSAID	28 (55%)		
Oral steroids (prednisolone)	16 (31%)		
Intraarticular steroids	32 (63%)		
*Conventional synthetic DMARDs:*			
Methotrexate Sulfasalazine	23 (45%) 4 (8%)		
*Biological DMARDs:*			
Adalimumab Etanercept	4 (8%) 4 (8%)		

*ANA* Antinuclear antibodies, *anti-CCP* Anti-cyclic citrullinated peptide antibody, *DMARDs* Disease modifying anti-rheumatic drugs, *ERA* enthesitis-related arthritis, *JIA* Juvenile idiopathic arthritis, *JPsA* Juvenile psoriatic arthritis, *N/A* Not applicable, *NSAID* Non-steroid anti-inflammatory drug, *RF* Rheumatoid factor

Values are shown as median (minimum – maximum) for continuous variables and numbers (percentage) for categorical variables

### Radiography of affected joints

Conventional radiography of affected joints was performed 0.4–17 years after disease onset in 47 (92%) out of 51 JIA patients. Radiographic investigations were not clinically motivated in 2 individuals with mild JIA, one individual declined the radiographic investigation, and one individual migrated and was therefore not available. Radiographic signs of joint erosions were detected in 15 individuals (32%) of which 3 had erosions at inclusion and 12 individuals developed erosions during follow-up. Joint erosions were mainly detected in the polyarticular (35%), ERA (100%), and JPsA (40%) subtypes, but only one individual with oligoarticular (5%) (Table 2).

**Table 2:** Detailed characteristics of the 15 patients with joint erosions

Number	Sex (F/M)	Age at inclusion (years)	JIA subtype	Anti-CCP (pos/neg)	RF (pos/neg)	Ongoing medication	Erosions detected (months from inclusion)
**1**	F	17.8	Polyarticular	pos	neg	Prednisolone, MTX, NSAID, i.a. corticosteroids	58
**2**	M	15.0	ERA	neg	neg	Prednisolone, MTX, i.a. corticosteroids	31
**3**	F	12.8	Polyarticular	pos	pos	MTX, NSAID, i.a. corticosteroids	19
**4**	M	15.3	Oligoarticular	pos	pos	NSAID	20
**5**	M	16.5	JPsA	neg	neg	Sulfasalazine, NSAID	4
**6**	M	17.7	ERA	neg	neg	i.a. corticosteroids	11
**7**	F	15.0	Undifferentiated	neg	neg	‒	47
**8**	M	15.3	ERA	neg	neg	Sulfasalazine, NSAID	–1
**9**	F	13.4	Polyarticular	neg	neg	Prednisolone, MTX, Etanercept	38
**10**	F	14.7	Oligoarticular	neg	neg	NSAID, i.a. corticosteroids	–16
**11**	F	14.8	Polyarticular	pos	pos	Prednisolone, MTX, i.a. corticosteroids	40
**12**	F	8.3	Polyarticular	neg	pos	Prednisolone, MTX, Adalimumab	39
**13**	F	16.5	Polyarticular	neg	neg	Prednisolone, MTX, i.a. corticosteroids	13
**14**	M	14.0	ERA	neg	neg	Prednisolone, Sulfasalazine, Adalimumab	28
**15**	M	14.9	JPsA	neg	neg	Prednisolone, MTX, NSAID	–1

*Anti-CCP* Anti-cyclic citrullinated peptide antibody, *ERA*, enthesitis-related arthritis, i.a. Intraarticular, *JIA* Juvenile idiopathic arthritis, *JPsA* Juvenile psoriatic arthritis, *MTX* Methotrexate, *NSAID* Non-steroid anti-inflammatory drug, *RF* Rheumatoid factor

### DXR-BMD and DXR Z-score

**Fig. 1:**
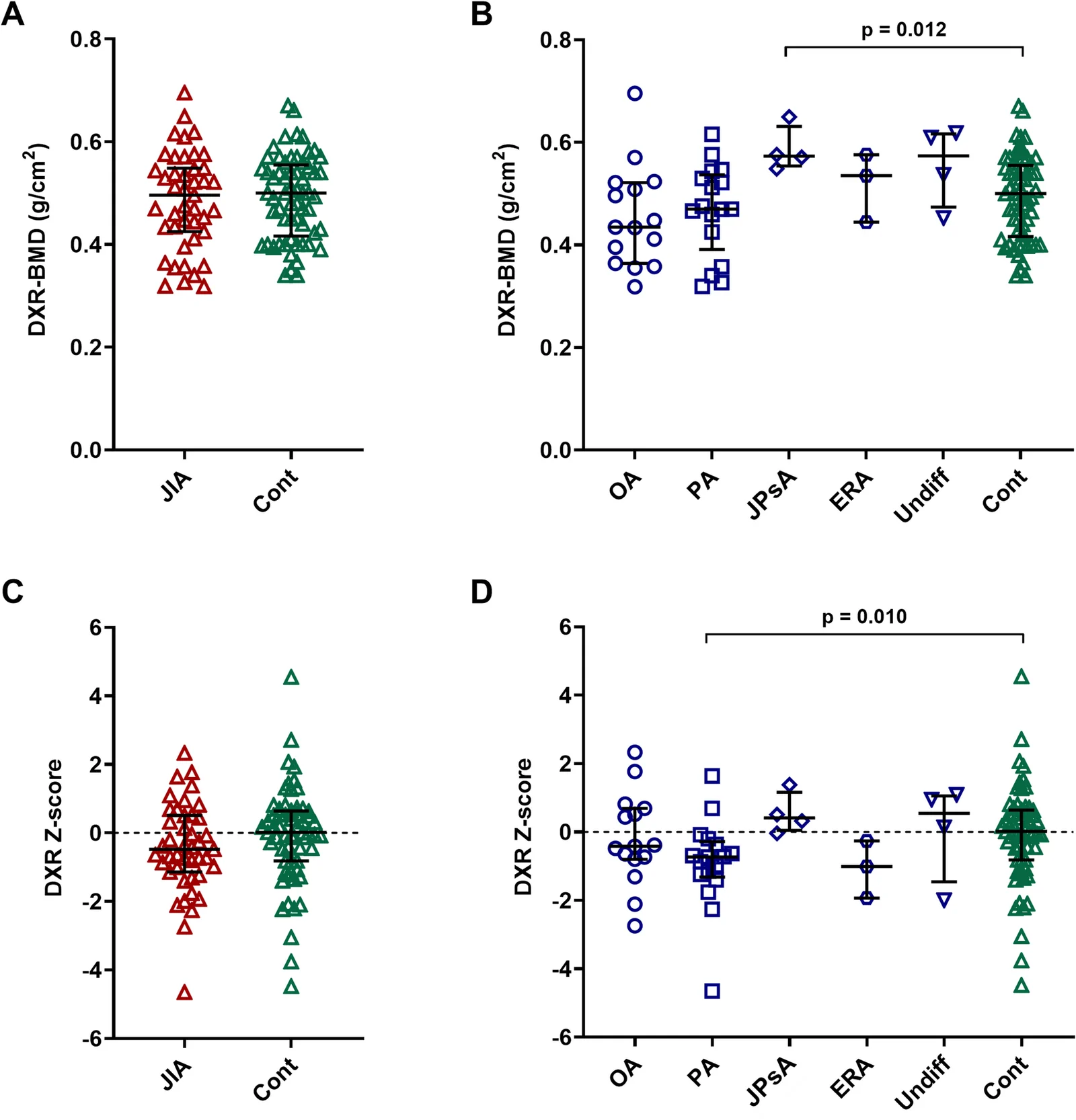
Median ± interquartile ranges for DXR-BMD and DXR Z-scores in the different JIA subtypes and control group. The polyarticular JIA group had lower DXR Z-score levels in comparison with the control group. JIA, *n* = 43; oligoarticular (OA), *n* = 15; polyarticular (PA), *n* = 17; juvenile psoriatic arthritis (JPsA), *n* = 4; enthesitis-related arthritis (ERA), *n* = 3; undifferentiated arthritis (Undiff), *n* = 4; controls (Cont), *n* = 61

Results of the DXR-BMD measurements are presented in Fig. 1A and B. Median (minimum–maximum) DXR-BMD values did not differ between the JIA group (*n* = 43) and control group (*n* = 61), 0.50 g/cm^2^ (0.32–0.70 g/cm^2^) and 0.50 g/cm^2^ (0.34–0.67 g/cm^2^), *p* = 0.746, respectively. Females with JIA (*n* = 25) had lower DXR-BMD values than males (*n* = 18), 0.47 g/cm^2^ (0.32–0.62 g/cm^2^) and 0.55 g/cm^2^ (0.36–0.70 g/cm^2^), *p* = 0.008, respectively. The JPsA group had higher DXR-BMD levels, 0.57 g/cm^2^ (0.55–0.65 g/cm^2^), in comparison with the control group, *p* = 0.012.

**Fig. 2:**
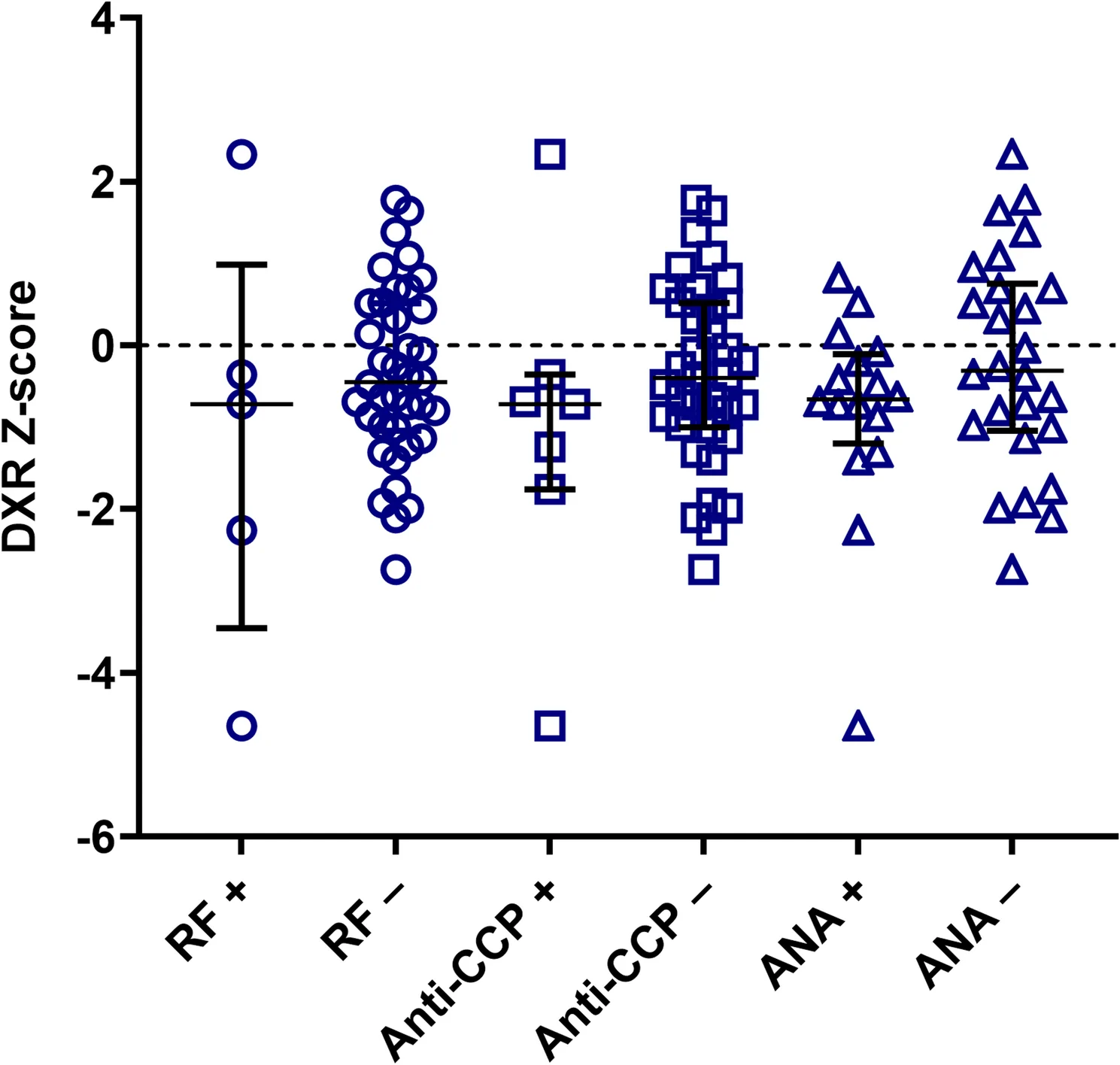
Median ± interquartile ranges for DXR Z-score in JIA positive and negative RF, anti-CCP and ANA markers

The DXR Z-scores did not differ between the JIA group (*n* = 43) and control group (*n* = 61), − 0.48 (–4.65 to 2.33) and 0.02 (–4.48 to 4.55), *p* = 0.126, respectively (Fig. 1C and D). Z-scores for DXR were lower in the polyarticular JIA group, − 0.73 (–4.65 to 1.64) in comparison with the control group, *p* = 0.010. No significant differences for DXR Z-scores were found for RF, anti-CCP and ANA positive individuals in comparison with RF, anti-CCP and ANA negative individuals (Fig. 2).

### Bone and cartilage biomarkers

**Fig. 3:**
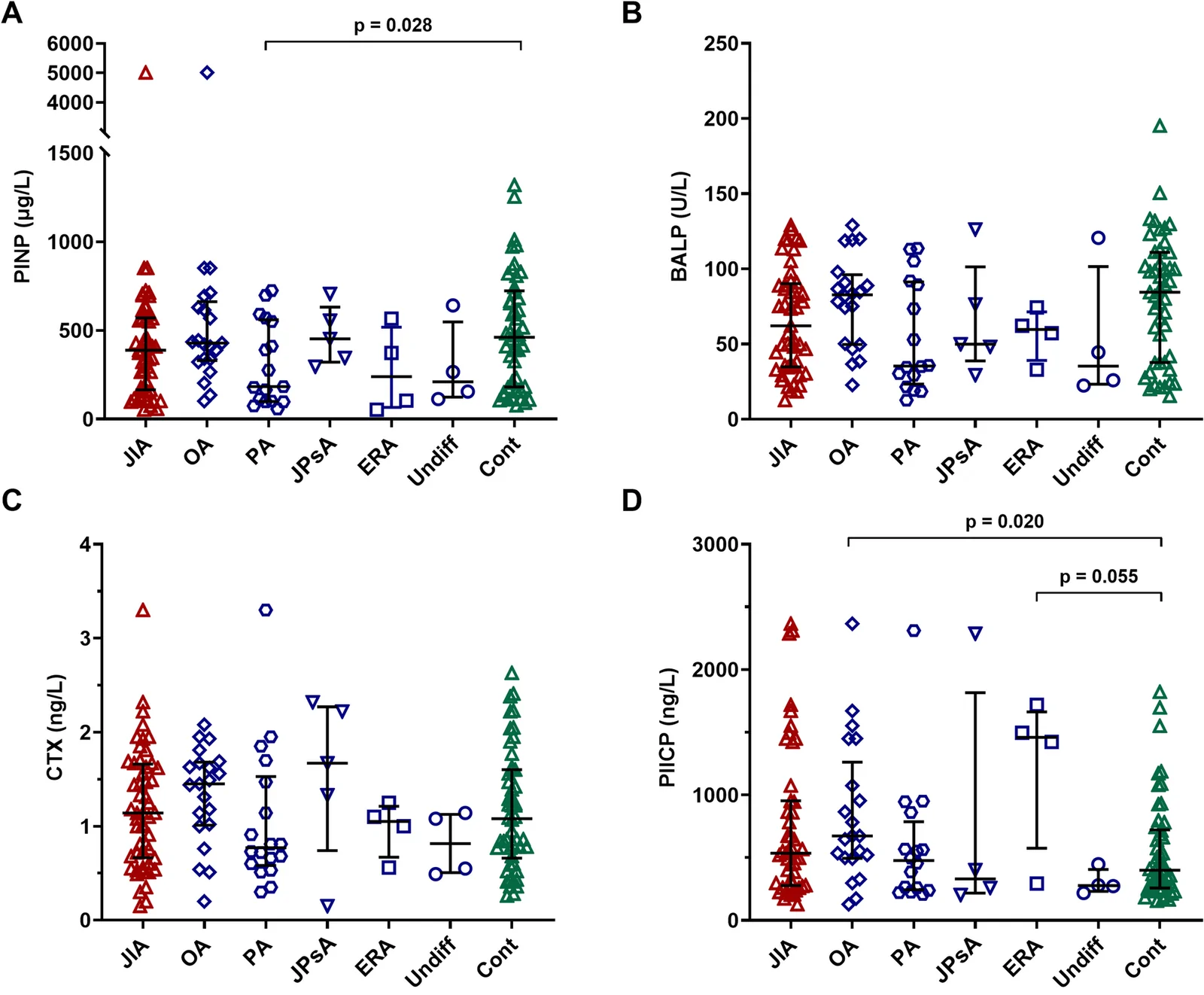
Serum levels of biomarkers with median ± interquartile range in the different JIA subtypes and in the control group. The polyarticular JIA group had lower PINP levels in comparison with the control group. In contrast, the PIICP levels were higher in the oligoarticular JIA group in comparison with the control group. **A)** PINP: JIA, *n* = 51; oligoarticular, *n* = 21; polyarticular, *n* = 17; JPsA, *n* = 5; ERA, *n* = 4; undifferentiated arthritis, *n* = 4; controls, *n* = 50. **B)** BALP: JIA, *n* = 49; oligoarticular, *n* = 20; polyarticular, *n* = 16; JPsA, *n* = 5; ERA, *n* = 4; undifferentiated arthritis, *n* = 4; controls, *n* = 47. **C)** CTX: JIA, *n* = 51; oligoarticular, *n* = 21; polyarticular, *n* = 17; JPsA, *n* = 5; ERA, *n* = 4; undifferentiated arthritis, *n* = 4; controls, *n* = 50. **D)** PIICP: JIA, *n* = 51; oligoarticular, *n* = 21; polyarticular, *n* = 17; JPsA, *n* = 5; ERA, *n* = 4; undifferentiated arthritis, *n* = 4; controls, *n* = 50

Baseline levels of PINP, BALP, CTX and PIICP in the JIA and control groups are presented in Fig. 3. The median (minimum–maximum) PINP levels did not differ between JIA and control groups, 388 µg/L (53–5011 µg/L) and 462 µg/L (77–2503 µg/L), *p* = 0.113, respectively. The polyarticular subtype JIA group had lower PINP levels, 183 µg/L (57–725 µg/L, in comparison with the control group at baseline, *p* = 0.028. Serum BALP did not differ between JIA and control groups, 62 U/L (13–129 U/L) and 84 U/L (16–195 U/L), *p* = 0.107, respectively. Nor did we observe any difference between the JIA and control groups for CTX, 1140 ng/L (150–3300 ng/L) and 1080 ng/L (260–2630 ng/L), *p* = 0.773, respectively. Serum PIICP did not differ between JIA and control groups, 552 ng/L (128–2366 ng/L) and 400 ng/L (156–1822 ng/L), *p* = 0.127, respectively. The oligoarticular subtype group had higher PIICP levels, 673 ng/L (128–2366 ng/L), in comparison with the control group at baseline, *p* = 0.020. A trend toward higher PIICP levels was observed in the ERA subtype group in comparison with the control group, *p* = 0.055. However, the ERA group comprised only 4 out of 51 JIA individuals limiting interpretation. When patients were stratified according to disease activity status, no significant differences were found in any of the investigated biomarkers between patients with active and inactive disease. Owing to the small number of patients with inactive disease (*n* = 5), the statistical power of these analyses was limited, and the results should be interpreted cautiously.

**Fig. 4:**
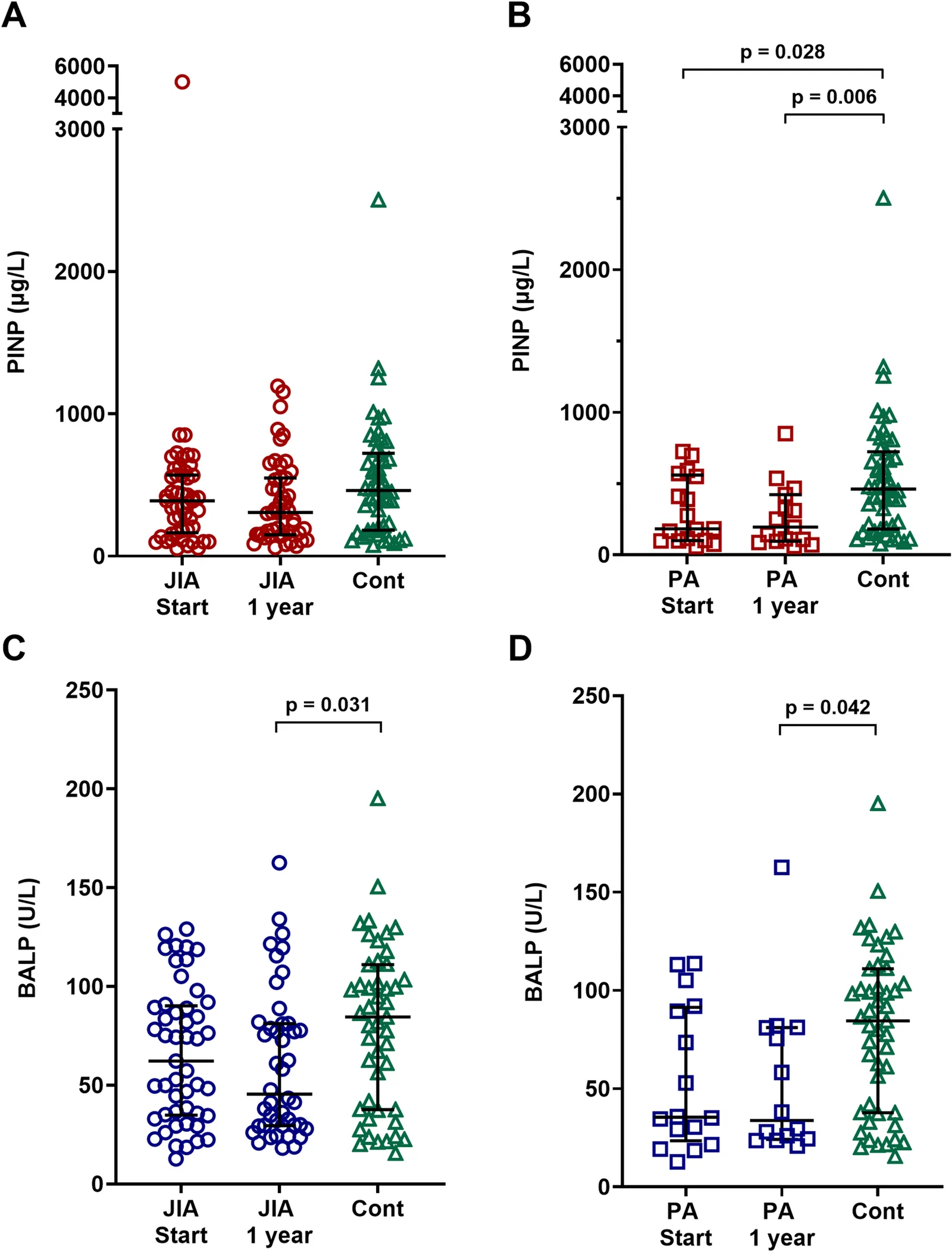
PINP and BALP with median ± interquartile range for all JIA individuals and for the polyarticular JIA group (Poly) at start and after 1 year, and controls. **A)** PINP: JIA at start, *n* = 51; JIA at 1 year, *n* = 48; controls, *n* = 50. **B)** PINP: Polyarticular at start, *n* = 17; JIA, 1 year, *n* = 15; controls, *n* = 50. **C)** BALP: JIA at start, *n* = 49; JIA at 1 year, *n* = 44; controls, *n* = 47. **D)** BALP: Polyarticular at start, *n* = 16; JIA, 1 year, *n* = 14; controls, *n* = 47

Figure 4 illustrates the bone formation markers PINP and BALP in the JIA and polyarticular subtype groups at baseline and after 1-year follow-up, in comparison with the control group. Serum PINP was lower in the polyarticular subtype group, both at baseline and after 1 year, in comparison with the control group, *p* = 0.028 and *p* = 0.006, respectively. Serum BALP was lower in both the JIA group and polyarticular subtype group after 1-year follow-up in comparison with the control group, *p* = 0.031 and *p* = 0.042, respectively.

Individuals treated with oral steroids (prednisolone) had lower CTX levels than those who had never received oral corticosteroids, 700 ng/L (150–1850 ng/L) vs. 1320 ng/L (200–3300 ng/L), respectively, *p* = 0.006. In contrast, levels of the other biomarkers (PINP, BALP, and PIICP) did not differ between individuals who had received oral steroids and those who had never received such treatment. None of the biomarkers showed significant differences regarding sex, active JIA disease, presence of joint erosions, or treatment with biological DMARDs. Furthermore, no biomarker was associated with the extent of observed joint erosions.

### Biomarkers in comparison with anti-CCP, RF, ANA and suPAR

No significant differences were found for the bone and cartilage biomarkers between positive and negative anti-CCP, RF and ANA individuals, and no significant correlations were found. Nor did we observe any significant associations between the suPAR levels and DXR-BMD, DXR Z-scores, and bone and cartilage biomarkers.

## Discussion

Childhood and adolescence are sensitive periods for bone mass accrual, with long-term implications for bone health later in life. In this prospective case–control study, we investigated bone mass and biomarkers of bone and cartilage turnover in a well‑characterized pediatric cohort with JIA. The principal findings were that children with polyarticular JIA had lower DXR Z-scores and lower levels of the bone formation markers PINP and BALP in comparison with the control group. In contrast, the bone resorption marker CTX did not differ between the JIA and control groups. Children with oligoarticular JIA had higher PIICP levels, indicating increased cartilage synthesis. No associations were observed between bone or cartilage biomarkers and anti‑CCP, RF, ANA, or suPAR.

Although overall DXR‑BMD values did not differ between the total JIA group and control group, the lower DXR Z‑scores in polyarticular subgroup indicate poor bone health. The lack of differences in absolute BMD values likely reflects age variability and the wide range of growth‑related bone mass among children. The use of BMD Z‑scores is therefore essential in pediatric populations [13, 16, 27]. A previous study reported a positive correlation between DXA and DXR absolute BMD values (g/m^2^) but no association between the corresponding Z-scores in individuals with JIA [28]. This contrasts with the findings of the current study; however, that study had a smaller sample size (*n* = 35 vs. 51) and a cross-sectional design, which may account for this discrepancy. Other studies have shown that bone density estimates by DXR is a feasible method for assessing BMD in pediatric populations, particularly in settings where DXA is not readily available and, furthermore, DXR has been approved by the U.S. Food and Drug Administration [12, 15–17]. The assessment of DXR was feasible, well‑tolerated, and practical in this study supporting its value as a cost‑effective modality for estimating BMD in children.

Roth et al. [29], used peripheral quantitative computed tomography to assess volumetric BMD in children with JIA and found reduced trabecular density in the polyarticular subgroup, while cortical density remained normal across all JIA subgroups. Polyarticular JIA is considered a more severe phenotype because it affects more joints than other subgroups leading to a higher risk of bone and cartilage destruction [30]. No associations with joint erosions were found in the current study; however, the number of patients with erosions detected was small (*n* = 15), limiting interpretation. Circulating bone and cartilage biomarkers may not adequately reflect local joint damage, which is largely driven by site-specific inflammatory processes. Similarly, suPAR, previously shown to be associated with joint erosions in JIA [25], did not correlate with DXR-BMD or any biomarker in this cohort, suggesting that suPAR may represent distinct aspects of JIA pathophysiology.

The reductions in the bone formation markers PINP and BALP observed in the polyarticular subgroup are consistent with previous, albeit limited, studies demonstrating altered bone remodeling in JIA in the context of chronic inflammation [23]. Pro-inflammatory cytokines such as tumor necrosis factor‑α, interleukin-1β, and interleukin‑6 disrupt the homeostatic balance of bone remodeling by suppressing osteoblastic bone formation while stimulating osteoclastogenesis [23, 31]. These cytokines also upregulate dickkopf-1 (DKK1), a secreted inhibitor of Wnt signaling that suppresses osteoblastogenesis [32], thereby further contributing to impaired bone formation [33, 34]. Elevated serum DKK1 levels have also been reported in HLA-B27-positive individuals with ERA, although these levels decreased significantly following treatment with DMARDs [35].

In contrast to PINP and BALP, CTX levels were not increased in our cohort, suggesting that systemic bone resorption is not substantially altered. Local bone erosions may therefore arise independently of detectable circulating biomarkers of resorption. Taken together, the reduced PINP and BALP levels suggest impaired bone accrual rather than increased bone resorption as the dominant mechanism contributing to reduced BMD Z-scores in children with JIA, particularly those with polyarticular disease.

Oral prednisolone treatment was associated with lower CTX levels, indicating suppressed bone resorption. In contrast, markers of bone formation and cartilage turnover i.e., (PINP, BALP, and PIICP) were not affected. Both steroid therapy and DMARDs reduce inflammatory activity. A lower degree of inflammation resulting from anti-inflammatory treatment may attenuate inflammation-induced bone degradation [36]. Glucocorticoids exert complex and time-dependent effects on bone remodeling including impaired osteoblast differentiation and function, and increased apoptosis of osteoblasts and osteocytes, which reduce bone formation and contribute to bone loss despite reduced bone resorption markers [37]. Therefore, the lower CTX levels observed should be interpreted cautiously and may reflect suppression of overall bone turnover rather than improved skeletal health.

The elevated PIICP levels in the oligoarticular subtype may reflect active cartilage matrix synthesis in joints with localized inflammation. This finding corresponds with the report by Winsz-Szczotka et al. [20] suggesting dynamic cartilage turnover early in the disease course. However, in that study, oligoarticular and polyarticular JIA were not analyzed separately, and additional subtypes were excluded. Overall, PIICP appears to capture aspects of cartilage biology distinct from markers of bone remodeling and may have potential as a complementary biomarker in JIA.

Several methodological strengths support the validity of this study, including its prospective design, well-defined clinical cohort investigated by the same pediatric rheumatologist, the use of DXR-BMD Z-scores, and the combined assessment of bone formation, bone resorption, and cartilage biosynthesis. Nonetheless, this study has several limitations. Sample sizes for certain JIA subtypes, particularly JPsA and ERA, were small, which reduced the power to detect subtype-specific differences. Nevertheless, this reflects the rarity of JIA and the inherent difficulties associated with assembling large pediatric cohorts with comprehensive clinical characterization. Although patients and controls were comparable with regard to age and sex, residual confounding by factors influencing bone turnover and BMD cannot be entirely excluded. Furthermore, the small subgroup sizes limited the statistical power to evaluate the potential confounding effects of age and treatment exposure, including oral glucocorticoid and DMARD use, on the observed biomarker findings. Information on cumulative steroid dose and treatment duration would have been valuable; however, these data were not collected. The absence of such information limited our ability to assess potential dose–response relationships related to steroid exposure in the 16 individuals (31%) receiving steroid treatment. Although the control group was carefully screened, radiographic evaluation beyond DXR was not performed, precluding comparisons of structural joint changes. In addition, Tanner staging, which influences bone turnover and bone accrual, was not systematically recorded and may have contributed to interindividual variability.

## Conclusion

Our findings have clinical implications for the management of children and adolescents with JIA, particularly those with polyarticular JIA who demonstrated impaired bone formation and reduced age‑adjusted bone mass. This indicates an increased risk of suboptimal peak bone mass acquisition and potentially fractures later in life. Longitudinal monitoring of bone health, together with early, targeted interventions, may therefore be especially important in this susceptible group to support optimal attainment of peak bone mass. Given the observed biomarkers profile, strategies aimed at increasing bone formation may be more appropriate than therapies that primarily inhibit bone resorption. Future research should include larger cohorts of well-defined JIA subtypes and integrate imaging modalities with comprehensive biomarker panels. Incorporating Tanner staging into such analyses will also be essential to accurately characterize bone health trajectories in children with JIA.

## Data Availability

The datasets used and/or analyzed during the current study are available and can be obtained from the corresponding author on reasonable request.

## Abbreviations

ANA: Antinuclear antibodies
Anti-CCP: Anti-cyclic citrullinated peptide antibodies
BALP: Bone-specific alkaline phosphatase
BMD: Bone mineral density
CRP: C-reactive protein
CTX: C-terminal telopeptide of type I collagen
CV: Coefficient of variation
DMARDs: Disease-modifying anti-rheumatic drugs
DXA: Dual-energy X-ray absorptiometry
DXR: Digital X-ray radiogrammetry
ELISA: Enzyme-linked immunosorbent assay
ERA: Enthesitis-related arthritis
ESR: Erythrocyte sedimentation rate
i.a.: intraarticular
JIA: Juvenile idiopathic arthritis
JPsA: Juvenile psoriatic arthritis
MTX: Methotrexate
NSAID: Non-steroid anti-inflammatory drug
PIICP: Type II procollagen C-terminal propeptide
PINP: Intact procollagen type I N-propeptide
RF: Rheumatoid factor
suPAR: soluble urokinase plasminogen activator receptor

## References

[1] Petty RE, Southwood TR, Manners P, Baum J, Glass DN, Goldenberg J, He X, Maldonado-Cocco J, Orozco-Alcala J, Prieur AM, et al. International League of Associations for Rheumatology classification of juvenile idiopathic arthritis: second revision, Edmonton, 2001. J Rheumatol. 2004;31(2):390–2. 14760812

[2] Zaripova LN, Midgley A, Christmas SE, Beresford MW, Baildam EM, Oldershaw RA. Juvenile idiopathic arthritis: from aetiopathogenesis to therapeutic approaches. Pediatr Rheumatol. 2021;19(1):135. 10.1186/s12969-021-00629-8. PMC8383464 34425842

[3] Kulyk M, Dzhus M. Factors associated with sarcopenia among young adults with juvenile idiopathic arthritis: a cross-sectional study. BMC Musculoskelet Disord. 2024;25(1):923. 10.1186/s12891-024-08051-3. PMC11572095 39558318

[4] Kulyk M, Westhovens R, De Vlam K, Lories R, Dzhus M. Bone health in young adults with inflammatory arthritis: assessment of associated factors of low bone mineral density. Arch Osteoporos. 2026;21(1). 10.1007/s11657-026-01710-z. 42053666

[5] Kulyk M, Yeremiya O, Westhovens R, Vlam K, Dzhus M. Muscle mass, strength, and sarcopenia in juvenile idiopathic arthritis: a scoping review. Pediatr Rheumatol. 2026;24(1):9. 10.1186/s12969-026-01188-6. PMC12918096 41580839

[6] Ravelli A, Martini A. Juvenile idiopathic arthritis. Lancet. 2007;369(9563):767–78. 10.1016/S0140-6736(07)60363-8. 17336654

[7] Ravelli A, Martini A. Early predictors of outcome in juvenile idiopathic arthritis. Clin Exp Rheumatol. 2003;21(5,Suppl31):S89–93. 14969057

[8] Van Dijkhuizen EHP, Wulffraat NM. Early predictors of prognosis in juvenile idiopathic arthritis: a systematic literature review. Ann Rheum Dis. 2015;74(11):1996–2005. 10.1136/annrheumdis-2014-205265. 24962873

[9] Van Rijn RR, Grootfaam DS, Lequin MH, Boot AM, van Beek RD, Hop WC, van Kuijk C. Digital radiogrammetry of the hand in a pediatric and adolescent Dutch Caucasian population: normative data and measurements in children with inflammatory bowel disease and juvenile chronic arthritis. Calcif Tissue Int. 2004;74(4):342–50. 10.1007/s00223-003-0020-x. 15255071

[10] Wilczek ML, Kälvesten J, Algulin J, Beiki O, Brismar TB. Digital X-ray radiogrammetry of hand or wrist radiographs can predict hip fracture risk – a study in 5,420 women and 2,837 men. Eur Radiol. 2013;23(5):1383–91. 10.1007/s00330-012-2706-9. PMC3622796 23229168

[11] Kulyk M, Grygorieva N, Musiienko A, Dzhus M. Bone mineral density, body composition, and muscle strength in young adults with juvenile idiopathic arthritis: a case–control study. BMC Rheumatol. 2026. 10.1186/s41927-026-00683-3. In Press.

[12] Rosholm A, Hyldstrup L, Bæcksgaard L, Grunkin M, Thodberg HH. Estimation of bone mineral density by digital X-ray radiogrammetry: theoretical background and clinical testing. Osteoporos Int. 2001;12(11):961–9. 10.1007/s001980170026. 11804024

[13] Lundestad A, Cetrelli L, Angenete OW, Augdal TA, Tylleskär K, Nordal EB, Rosendahl K, Börmark Hoftun G, Hoff M, Romundstad PR, Rygg M. Bone health in juvenile idiopathic arthritis compared with controls based on a Norwegian observational study. RMD Open. 2025;11(2). 10.1136/rmdopen-2025-005605. PMC12182186 40537274

[14] Leijten AD, Hampsink B, Janssen M, Klein WM, Draaisma JMT. Can digital X-ray radiogrammetry be an alternative for dual-energy X-ray absorptiometry in the diagnosis of secondary low bone quality in children? Eur J Pediatr. 2019;178(9):1433–41. 10.1007/s00431-019-03425-5. PMC6694095 31352546

[15] Mergler S, de Man SA, Boot AM, Heus KG, Huijbers WA, van Rijn RR, Penning C, Evenhuis HM. Automated radiogrammetry is a feasible method for measuring bone quality and bone maturation in severely disabled children. Pediatr Radiol. 2016;46(7):1017–22. 10.1007/s00247-016-3548-4. PMC4883271 27026024

[16] Augdal T, Angenete O, Zadig P, Lundestad A, Nordal E, Shi X, Rosendahl K. The assessment of bone health in children with juvenile idiopathic arthritis; comparison of different imaging-based methods. Pediatr Rheumatol. 2024;22(1):80. 10.1186/s12969-024-01018-7. PMC11363637 39210351

[17] Guerri S, Mercatelli D, Aparisi Gomez MP, Napoli A, Battista G, Guglielmi G, Bazzocchi A. Quantitative imaging techniques for the assessment of osteoporosis and sarcopenia. Quant Imaging Med Surg. 2018;8(1):60–85. 10.21037/qims.2018.01.05. PMC5835658 29541624

[18] Koivula M-K, Risteli L, Risteli J. Measurement of aminoterminal propeptide of type I procollagen (PINP) in serum. Clin Biochem. 2012;45(12):920–7. 10.1016/j.clinbiochem.2012.03.023. 22480789

[19] Bonde M, Garnero P, Fledelius C, Qvist P, Delmas PD, Christiansen C. Measurement of bone degradation products in serum using antibodies reactive with an isomerized form of an 8 amino acid sequence of the C-telopeptide of type I collagen. J Bone Min Res. 1997;12(7):1028–34. 10.1359/jbmr.1997.12.7.1028. 9200001

[20] Winsz-Szczotka K, Kuznik-Trocha K, Lachor-Motyka I, Lemski W, Olczyk K. Concerted actions by PIICP, CTXII, and TNF-alpha in patients with juvenile idiopathic arthritis. Biomolecules. 2021;11(5):648. 10.3390/biom11050648. PMC8146247 33924892

[21] Ladang A, Rauch F, Delvin E, Cavalier E. Bone turnover markers in children: from laboratory challenges to clinical interpretation. Calcif Tissue Int. 2023;112(2):218–32. 10.1007/s00223-022-00964-2. 35243530

[22] Schini M, Vilaca T, Gossiel F, Salam S, Eastell R. Bone turnover markers: basic biology to clinical applications. Endocr Rev. 2023;44(3):417–73. 10.1210/endrev/bnac031. PMC10166271 36510335

[23] Di Marcello F, Di Donato G, d’Angelo DM, Breda L, Chiarelli F. Bone health in children with rheumatic disorders: focus on molecular mechanisms, diagnosis, and management. Int J Mol Sci. 2022;23(10):5725. 10.3390/ijms23105725. PMC9143357 35628529

[24] Smith EMD, Foster HE, Beresford MW. Adding to complexity: comorbidity in paediatric rheumatic disease. Rheumatology. 2013;52(1):22–33. 10.1093/rheumatology/kes256. 23024018

[25] Lewander P, Wirestam L, Dahle C, Wetterö J, Sjöwall C. Serum levels of soluble urokinase plasminogen activator receptor in juvenile idiopathic arthritis: a single-center Swedish case-control study. Pediatr Rheumatol. 2023;21(1):49. 10.1186/s12969-023-00832-9. PMC10226192 37246218

[26] Wallace CA, Giannini EH, Huang B, Itert L, Ruperto N. American College of Rheumatology provisional criteria for defining clinical inactive disease in select categories of juvenile idiopathic arthritis. Arthritis Care Res. 2011;63(7):929–36. 10.1002/acr.20497. 21717596

[27] Shuhart CR, Yeap SS, Anderson PA, Jankowski LG, Lewiecki EM, Morse LR, Rosen HN, Weber DR, Zemel BS, Shepherd JA. Executive summary of the 2019 ISCD position development conference on monitoring treatment, DXA cross-calibration and least significant change, spinal cord injury, peri-prosthetic and orthopedic bone health, transgender medicine, and pediatrics. J Clin Densitom. 2019;22(4):453–71. 10.1016/j.jocd.2019.07.001. 31400968

[28] Nusman CM, Anink J, Otten MH, van Rossum MAJ, van Rijn RR, Maas M, van Suijlekom-Smit LWA. Bone health of patients with juvenile idiopathic arthritis: a comparison between dual-energy X-ray absorptiometry and digital X-ray radiogrammetry. Eur J Radiol. 2015;84(10):1999–2003. 10.1016/j.ejrad.2015.06.015. 26116967

[29] Roth J, Palm C, Scheunemann I, Ranke MB, Schweizer R, Dannecker GE. Musculoskeletal abnormalities of the forearm in patients with juvenile idiopathic arthritis relate mainly to bone geometry. Arthritis Rheum. 2004;50(4):1277–85. 10.1002/art.20128. 15077312

[30] Oberle EJ, Harris JG, Verbsky JW. Polyarticular juvenile idiopathic arthritis – epidemiology and management approaches. Clin Epidemiol. 2014;6:379–93. 10.2147/CLEP.S53168. PMC4216020 25368531

[31] Coury F, Peyruchaud O, Machuca-Gayet I. Osteoimmunology of bone loss in inflammatory rheumatic diseases. Front Immunol. 2019;10:679. 10.3389/fimmu.2019.00679. PMC6456657 31001277

[32] Bafico A, Liu G, Yaniv A, Gazit A, Aaronson SA. Novel mechanism of Wnt signalling inhibition mediated by dickkopf-1 interaction with LRP6/Arrow. Nat Cell Biol. 2001;3(7):683–6. 10.1038/35083081. 11433302

[33] Schett G, Stach C, Zwerina J, Voll R, Manger B. How antirheumatic drugs protect joints from damage in rheumatoid arthritis. Arthritis Rheum. 2008;58(10):2936–48. 10.1002/art.23951. 18821703

[34] Redlich K, Smolen JS. Inflammatory bone loss: pathogenesis and therapeutic intervention. Nat Rev Drug Discov. 2012;11(3):234–50. 10.1038/nrd3669. 22378270

[35] Ye L, Jin Y, Huang H, Ding F, Xu X, Liu C, Bao S, Wu J, Jin Y. The plasma levels of dickkopf-1 elevated in patients with juvenile idiopathic arthritis. Pediatr Res. 2023;94(4):1349–54. 10.1038/s41390-023-02637-8. 37179434

[36] Weinstein RS. Glucocorticoid-induced bone disease. N Engl J Med. 2011;365(1):62–70. 10.1056/NEJMcp1012926. 21732837

[37] Gado M, Baschant U, Hofbauer LC, Henneicke H. Bad to the bone: the effects of therapeutic glucocorticoids on osteoblasts and osteocytes. Front Endocrinol. 2022;13:835720. 10.3389/fendo.2022.835720. PMC9008133 35432217

